# Evaluating the Therapeutic Mechanisms of Selected Active Compounds in *Houttuynia cordata* Thunb. in Pulmonary Fibrosis *via* Network Pharmacology Analysis

**DOI:** 10.3389/fphar.2021.733618

**Published:** 2021-09-30

**Authors:** De-Wei Zhu, Qun Yu, Ji-Jia Sun, Yun-Hui Shen

**Affiliations:** School of Pharmacy, Shanghai University of Traditional Chinese Medicine, Shanghai, China

**Keywords:** pulmonary fibrosis, *Houttuynia cordata* Thunb., molecular mechanism, network pharmacology, signaling pathway analysis

## Abstract

Pulmonary fibrosis, a common outcome of pulmonary interstitial disease of various different etiologies, is one of the most important causes of respiratory failure. *Houttuynia cordata* Thunb. (family: Saururaceae) (*H. cordata*), as has been reported, is a Chinese herbal medicine commonly used to treat upper respiratory tract infection and bronchitis. Our previous study has proven that sodium houttuyfonate (an additional compound from sodium bisulfite and houttuynin) had beneficial effects in the prevention of pulmonary fibrosis (PF) induced by bleomycin (BLM) in mice. In the present study, network pharmacology was used to investigate the efficiency and potential mechanisms of *H. cordata* in PF treatment. Upon manual collection from the literature and databases such as TCMSP and TCM-ID, 10 known representative ingredients of *H. cordata* species were screened. Then, the prediction of the potential active ingredients, action targets, and signaling pathways were conducted through the Gene Ontology (GO), protein–protein interaction (PPI),and Kyoto Encyclopedia of Genes and Genomes (KEGG) enrichment analyses. The results of network pharmacology prediction suggested that *H. cordata* may act through multiple signaling pathways to alleviate PF, including the phosphatidylinositol 3-kinase-protein kinase B (PI3K/AKT) pathways, mitogen-activated protein kinase (MAPK) pathways, the tumor necrosis factor (TNF) pathways, and interleukin-17 (IL-17) signaling pathways*.* Molecular docking experiments showed that the chemical constituents of *H. cordata* had good affinity with TNF, MAPK1, and AKT1, and using lipopolysaccharide (LPS)-induced A549 cells, a model was established to verify the anti-pulmonary fibrosis effects and related mechanisms of *H. cordata*–relevant constituents. Finally, these evidences collectively suggest *H. cordata* may alleviate PF progression *via* PI3K/Akt, MAPK, and TNF signaling pathways and provide novel insights to verify the mechanism of *H. cordata* in the treatment of PF.

## Introduction

Pulmonary fibrosis (PF) is a chronic, progressive, and devastating interstitial lung disease mainly resulting from toxic insults, autoimmune injuries, drug-induced injuries, infectious injuries, or traumatic injuries. As can be affected by age, genetic predisposing factors, and environmental exposures, PF produces many different pathological types, which are mainly classified as idiopathic pulmonary fibrosis (IPF) and fibrosing alveolitis. PF is mainly characterized by diffuse pneumonia in the early period, secreting pro-inflammatory cytokines such as the tumor necrosis factor α (TNF-α), interleukin-1β (IL-1β), and IL-6. Alveolar epithelial cell (AEC) injury and excessive deposition of collagen in extracellular matrix (ECM) ultimately result in progressive scarring and loss of lung function (Wynn and Vannella, 2016). With approximately 3 million people being affected worldwide, as shown in the related literature, IPF has an increasing burden (George et al., 2020). At present, there is no effective means or drugs that can ameliorate PF except lung transplantation in patients with a median survival of 3–5 years from the time of diagnosis (Meyer, 2017).‬‬‬‬‬‬‬‬‬‬‬‬

PF, with clinical manifestations such as progressive dyspnea, cough, sputum, chest pain, vomiting, dry mouth, dry throat, shortness of breath, and fatigue, was defined as “lung impediment” or “lung wilting” in books of traditional Chinese medicine, and its syndrome types were divided into lung–kidney Qi deficiency (Fei shen qi xu), dual deficiency of the lung and spleen (Pi fei liang xu), Qi deficiency with blood stasis (Qi xu xue yu), and phlegm obstructing the vessels and collaterals (Tan zu mai luo). They were mainly treated by methods of invigorating the lungs and kidneys (Bu fei yi shen), fortifying the spleen and boosting the lung (Jian pi yi fei), promoting blood circulation and dredging collaterals (Huo xue tong luo), transforming dampness and dispelling phlegm (Hua shi qu tan), and harmonizing and percolating dampness (He jie shen shi). The treatment process used decoctions including Schisandra decoction, Bufei decoction, and Buyang Huanwu decoction , and heat-clearing and toxin-resolving Chinese medicinal materials such as *Centella asiatica* (L.) Urban, *Ophiopogon japonicus* (L.f.) Ker-Gawl., and *Houttuynia cordata* Thunb. (family: Saururaceae) (*H. cordata*). The traditional Chinese medicine (TCM) mainly produces synergistic effects *via* the synergistic mechanism of actions with the active ingredients, enhancing functions, and producing less toxicity, which makes enormous contributions to modern drug development and disease treatment. Some active ingredients in the traditional Chinese medicine play a good role in the progression of PF, just like paclitaxel (PTX) (Wang et al., 2013), tanshinone IIA (Tan IIA) (He et al., 2015), and andrographolide (Andro) (Guo et al., 2016).

*H. cordata*, famous in China, Japan, Korea, and Northeast India for its medicinal properties, is used in the treatment of a number of diseases such as pneumonia, diabetes, and even SARS (Lau et al., 2008). Studies have found that *H. cordata* also has a certain inhibitory effect on PF; for instance, the volatile oil of *H. cordata* contains 4-terpineol, *α*-terpineol, l-bornyl acetate, and methyl-n-nonyl ketone, and has good anti-inflammatory and anti-pulmonary fibrosis effects *in vitro* and *in vivo* (Du et al., 2012). The aqueous extract of *H. cordata* has effects of anti-pulmonary fibrosis by inhibiting the expression of superoxide dismutase, malondialdehyde, hydroxyproline, interferon-γ (IFN-γ), and TNF-α (Ng et al., 2007). Qing fei xie ding granules (Sun et al., 2018), mainly including *Ephedra sinica*, apricot kernels, and *H. cordata*, have a protective effect on PF as a novel Chinese traditional patent medicine. However, the anti-pulmonary fibrosis mode and the mechanism of action of *H. cordata* are not fully clarified. To address the protective effects of *H. cordata* on PF, our previous study has shown that sodium houttuyfonate is an attractive candidate alleviating IPF and pulmonary toxicity induced by BLM through downregulation of TGFβ1-Smad2/3 signal transduction (Shen et al., 2021).

Despite the obvious medicinal effects and clinical uses of TCM, little has been elucidated regarding the underlying molecular mechanisms. Network pharmacology integrates several disciplines, including systems biology, network biology, computational biology, multi-target pharmacology, and molecular pharmacology. And the core is the construction of a drug molecule–target–disease link network and the systematic analysis of the three relationships. It can be used to predict active molecules and actions of TCM, and then to search for new pharmacological mechanisms of action and broaden the indications of TCM, *etc*. Molecular docking is used to design drugs by *in silico* modeling of the interaction of target proteins with drug molecules. Molecular docking has been gradually applied to reveal the relevant mechanism of action of drugs in the development of new drugs. This study aims to utilize network pharmacology and molecular docking to seek the active compounds of *H. cordata*, construct the drug–compounds–genes–disease network, and to analyze the underlying mechanism of *H. cordata* in the treatment of PF.

## Methods

### Main Candidate Active Ingredients and Targets of *H. cordata*

The keyword “Houttuyniae Herba” was searched in TCMSP (traditional Chinese medicine ystems pharmacology database and analysis platform, http://tcmspw.com/tcmsp.php) and TCM-ID (traditional Chinese medicine integrated database, http://www.megabionet.org/tcmid/). Active ingredients were retrieved from them. The chemical information of main active ingredients was collected from the PubChem database (https://pubchem.ncbi.nlm.nih.gov/) and the Canonical SMILES of all ingredients were traced back to PubChem (http://pubchem.ncbi.nih.gov). The major components in *H. cordata* were obtained by a literature review and screening of public databases. The potential therapeutic targets of the main active components were predicted by matching them in the TCMSP, SEA (https://sea.bkslab.org/), HitPick (http://mips.helmholtz-muenchen.de/hitpick/), SwissTargetPrediction (http://www.swisstargetprediction.ch/), and STITCH (http://stitch.embl.de/) tools. Among them, according to TC > 0.4 in SEA, according to precision >50% SwissTargetPrediction in HitPick, the top 15 with the highest scores were selected, and according to score >0.4 in stitch.

### Mining of Pulmonary Fibrosis–Related Targets

With “pulmonary fibrosis” as the keyword, DisGeNET (https://www.disgenet.org/), GeneCards (https://www.genecards.org/), and OMIM (https://www.omim.org/) potential disease target analysis platforms were utilized to search for human genes related to PF. After data deduplication/integration, intersecting genes were obtained and considered as therapeutic targets relevant to PF.

### Drug–Target–Disease Network Construction

Active ingredients of *H. cordata*–related targets and PF-related targets were imported into the Venn online tool (http://www.bioinformatics.com.cn/) to obtain the intersection and a Venn diagram, in which potential targets of *H. cordata* for the treatment of PF were revealed. The above potential target gene ID was identified and standardized through the UniProt database (https://www.uniprot.org). The target network of *H. cordata* major ingredients acting on PF was analyzed by Cytoscape 3.7.1 built-in tools.

### Protein–Protein Interaction Network Analysis

To determine the key *H. cordata* targets, common targets were used to build a PPI network using the STRING database (https://string-db.org/) and Cytoscape software. And the core target proteins were identified in the PPI network.

### Go Analysis and KEGG Pathway Enrichment Analysis

The overlapped targets were further analyzed by the GO function analysis and KEGG enrichment analysis using the clusterProfiler package based on R language. The filtering thresholds for the retrieved results were *p* < 0.05 and q < 0.05.

### Molecular Docking Simulation

The binding ability of key components and key targets was verified to explore the accurate binding modes through molecular docking simulation. The PDB database (http://www.rcsb.org/) and the optimal models visualized using PyMOL (2.0) software (http://www.pymol.org/2/) and AutoDockVina software (http://vina.scripps.edu/) were used to perform the molecular docking of active components and core targets.

## Experiment Validation

### Materials and Methods

#### Chemicals and Regents

Sodium new houttuyfonate (SNH, MW: 330.41, purity ≥ 98%), quercetin (MW: 302.24, purity ≥ 97%), and 2-undecanone (MW: 170.29, purity ≥ 99%) were purchased from Shanghai Yuanye Biotechnology Co., Ltd. (Shanghai, China).

#### Cell Culture

A549 cells, obtained from the Cell Bank of the Chinese Academy of Sciences, were cultured in DMEM-H with 10% fetal bovine serum (Gibco, United States), 100 U/mL penicillin, and 100 ng/ml streptomycin with 5% CO_2_ at 37°C. The EMT model of lipopolysaccharide (LPS)-induced A549 was reproduced in our study (Cui et al., 2019). For the EMT model, when the A549 cells reached 70–80% confluence on 6-well tissue culture plates, they were placed in DMEM-H supplemented with 1% fetal bovine serum for 12 h. Subsequently, the culture was replaced to DMEM-H with 2% fetal bovine serum and then treated with 10 μg/ml LPS (from *Escherichia coli* 0111: B4, Sigma-Aldrich, St. Louis, MO, United States) and SNH, quercetin, and 2-undecanone alone or in combination for up to 24 h, respectively.

#### Western Blot Analyses

The cells were lysed in RIPA lysis buffer (Beyotime, China) with 1% phenylmethylsulfonylfluoride (PMSF, Beyotime, China), 2% phosphorylated proteinase inhibitor (Beyotime, China), and 2% protease inhibitor (Beyotime, China). And 30 μg of the protein was separated using sodium dodecyl sulfate polyacrylamide gel electrophoresis (SDS-PAGE) (10%), and then was transferred onto a PVDF membrane. After blocking, the membranes were incubated overnight at 4°C with the primary antibody against β-actin (1:2,000, Affinity, United States), Phospho-AKT (1:1,000, Proteintech, United States), AKT (1:1,000, CST, United States), p44/42 MAPK (ERK1/2) (1:1,000, CST, United States of America), Phospho-p44/42 MAPK (ERK1/2) (1:1,000, CST, United States), Phospho-p38 MAPK (1:1,000, CST, United States), and TNF-α (1:1,000, CST, United States). Next, membranes were washed in TBS-T several times, and then were incubated for 2 h in horseradish peroxidase (HRP)-linked anti-rabbit (1:2,000, CST, United States) or anti-mouse (1:2,000, CST, United States) at room temperature. After washing with TBS-T again, the protein bands were analyzed using ChemiScope 3500mini exposure instrument (Clinx Science Instruments Co., Ltd. Shanghai, China) with a chemiluminescence substrate.

#### Statistical Analysis

All values were expressed as mean ± standard deviation (SD), and data and analyses for graphing were processed with SPSS 25.0 statistical software and GraphPad Prism 8.0.2 software. Comparisons between groups were performed using one-way ANOVA. Values of *p* < 0.05 were considered statistically significant.

## Results

**FIGURE 1:**
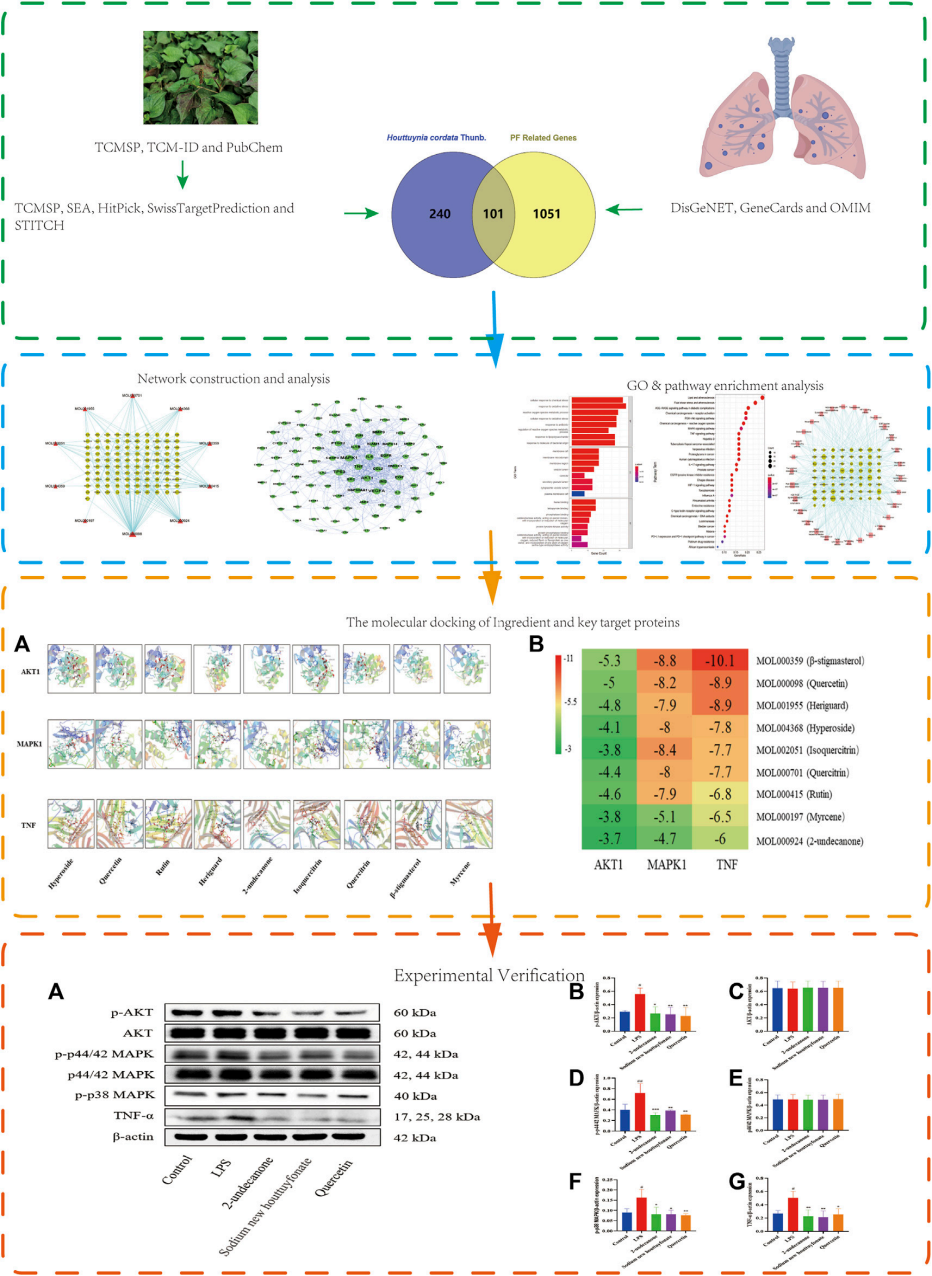
Flowchart of network pharmacology research on the mechanism of *H. cordata* in anti-pulmonary fibrosis.

This study reveals the mechanism of the anti-pulmonary fibrosis pharmacological action of *H. cordata* by a TCM network pharmacology–based strategy and provides ideas for *H. cordata* for further drug development. This workflow is shown in Figure 1.

### *H. cordata*’s Active Ingredients and Potential Target Prediction Results

*H. cordata*’s chemical constituents were collected from TCMSP. A total of 10 main components were candidated after removing the redundant ones from the literature and public databases. The 10 components were as follows: hyperoside, quercetin, rutin, heriguard, houttuynin, 2-undecanone, isoquercitrin, quercitrin, β-stigmasterol, and myrcene (Table 1). A total of 341 targets for the 10 chemical compositions were obtained after gene standardization and the elimination of repetitive values.

**TABLE 1:** Main chemical constituents of *H. cordata*.

Name	MOL	Formula	MW	TPSA	Silicos-IT LogSw	Silicos-IT class	GI absorption	Lipinski	Bioavailability score	Synthetic accessibility
Hyperoside	MOL004368	C_21_H_20_O_12_	464.38	210.51	−1.51	Soluble	Low	2	0.17	5.32
Quercetin	MOL000098	C_15_H_10_O_7_	302.24	131.36	−3.24	Soluble	High	0	0.55	3.23
Rutin	MOL000415	C_27_H_30_O_16_	610.52	269.43	−0.29	Soluble	Low	3	0.17	6.52
Heriguard	MOL001955	C_16_H_18_O_9_	354.31	164.75	0.4	Soluble	Low	1	0.11	4.16
Houttuynin	MOL004359	C_12_H_22_O_2_	198.3	34.14	−3.81	Soluble	High	0	0.55	1.76
2-Undecanone	MOL000924	C_11_H_22_O	170.29	17.07	−3.83	Soluble	High	0	0.55	1.72
Isoquercitrin	MOL002051	C_21_H_20_O_12_	464.38	210.51	−1.51	Soluble	Low	2	0.17	5.31
Quercitrin	MOL000701	C_21_H_20_O_11_	448.38	190.28	−2.08	Soluble	Low	2	0.17	5.28
β-Stigmasterol	MOL000359	C_29_H_50_O	414.71	20.23	−6.19	Poorly soluble	Low	1	0.55	6.3
Myrcene	MOL000197	C_10_H_16_	136.23	0	−2.42	Soluble	Low	0	0.55	2.85

### Feature Analysis of Active Compound–Drug Target Network

**FIGURE 2:**
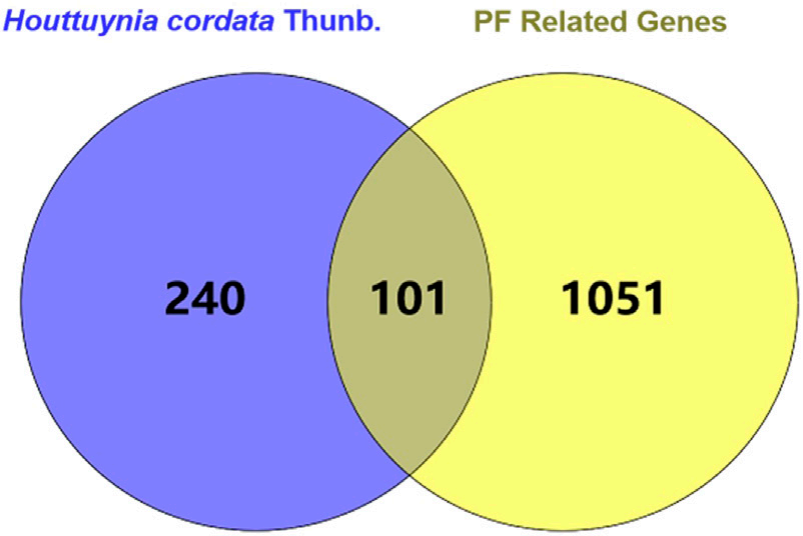
Venn diagram of common gene targets of pulmonary fibrosis and *H. cordata*.

**FIGURE 3:**
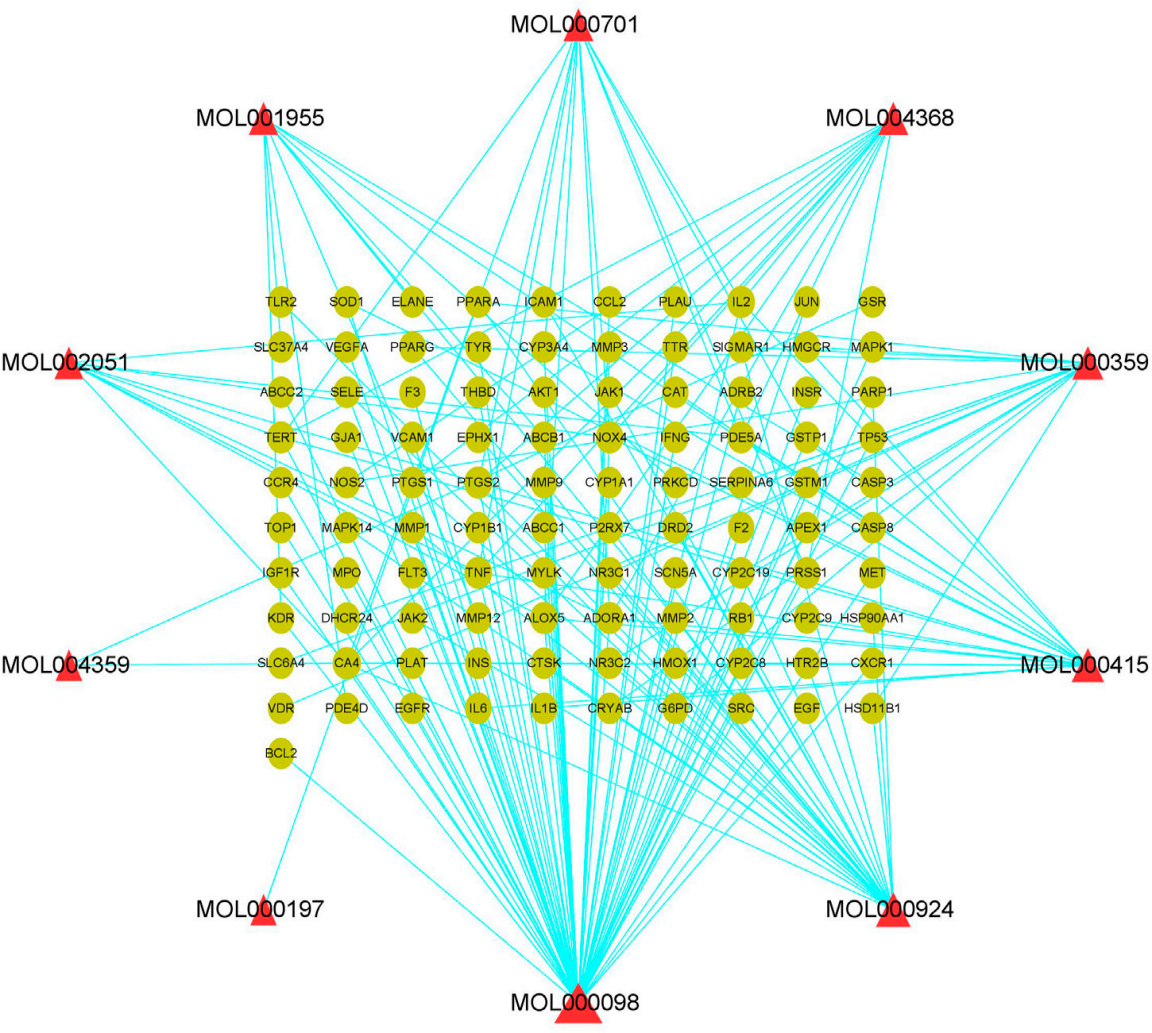
Component target network of 10 active ingredients of *H. cordata* against pulmonary fibrosis, in which triangles represent active ingredients and dots are action targets.

PF-related targets were collected from DisGeNET, GeneCards, OMIM, and Drugbank databases. After merging and deleting duplicate values, a total of 1,152 targets were obtained. As shown in Figure 2, 101 predicted targets were obtained after the intersection of drug-related targets and PF-related targets. The details of common target genes are shown in Table 2. 101 therapeutic targets were confirmed by the UniProt database, and a predicted target network of *H. cordata*'s major ingredient’s actions on PF was constructed using Cytoscape software (Figure 3).

**TABLE 2:** Shared hub targets between *H. cordata* and pulmonary fibrosis.

Number	Gene name	Number	Gene name	Number	Gene name
1	ABCB1	36	GSR	71	PARP1
2	ABCC1	37	GSTM1	72	PDE4D
3	ABCC2	38	GSTP1	73	PDE5A
4	ADORA1	39	HMGCR	74	PLAT
5	ADRB2	40	HMOX1	75	PLAU
6	AKT1	41	HSD11B1	76	PPARA
7	ALOX5	42	HSP90AA1	77	PPARG
8	APEX1	43	HTR2B	78	PRKCD
9	BCL2	44	ICAM1	79	PRSS1
10	CA4	45	IFNG	80	PTGS1
11	CASP3	46	IGF1R	81	PTGS2
12	CASP8	47	IL1B	82	RB1
13	CAT	48	IL2	83	SCN5A
14	CCL2	49	IL6	84	SELE
15	CCR4	50	INS	85	SERPINA6
16	CRYAB	51	INSR	86	SIGMAR1
17	CTSK	52	JAK1	87	SLC37A4
18	CXCR1	53	JAK2	88	SLC6A4
19	CYP1A1	54	JUN	89	SOD1
20	CYP1B1	55	KDR	90	SRC
21	CYP2C19	56	MAPK1	91	TERT
22	CYP2C8	57	MAPK14	92	THBD
23	CYP2C9	58	MET	93	TLR2
24	CYP3A4	59	MMP1	94	TNF
25	DHCR24	60	MMP12	95	TOP1
26	DRD2	61	MMP2	96	TP53
27	EGF	62	MMP3	97	TTR
28	EGFR	63	MMP9	98	TYR
29	ELANE	64	MPO	99	VCAM1
30	EPHX1	65	MYLK	100	VDR
31	F2	66	NOS2	101	VEGFA
32	F3	67	NOX4		
33	FLT3	68	NR3C1		
34	G6PD	69	NR3C2		
35	GJA1	70	P2RX7		

### PPI Network Analysis Results

**FIGURE 4:**
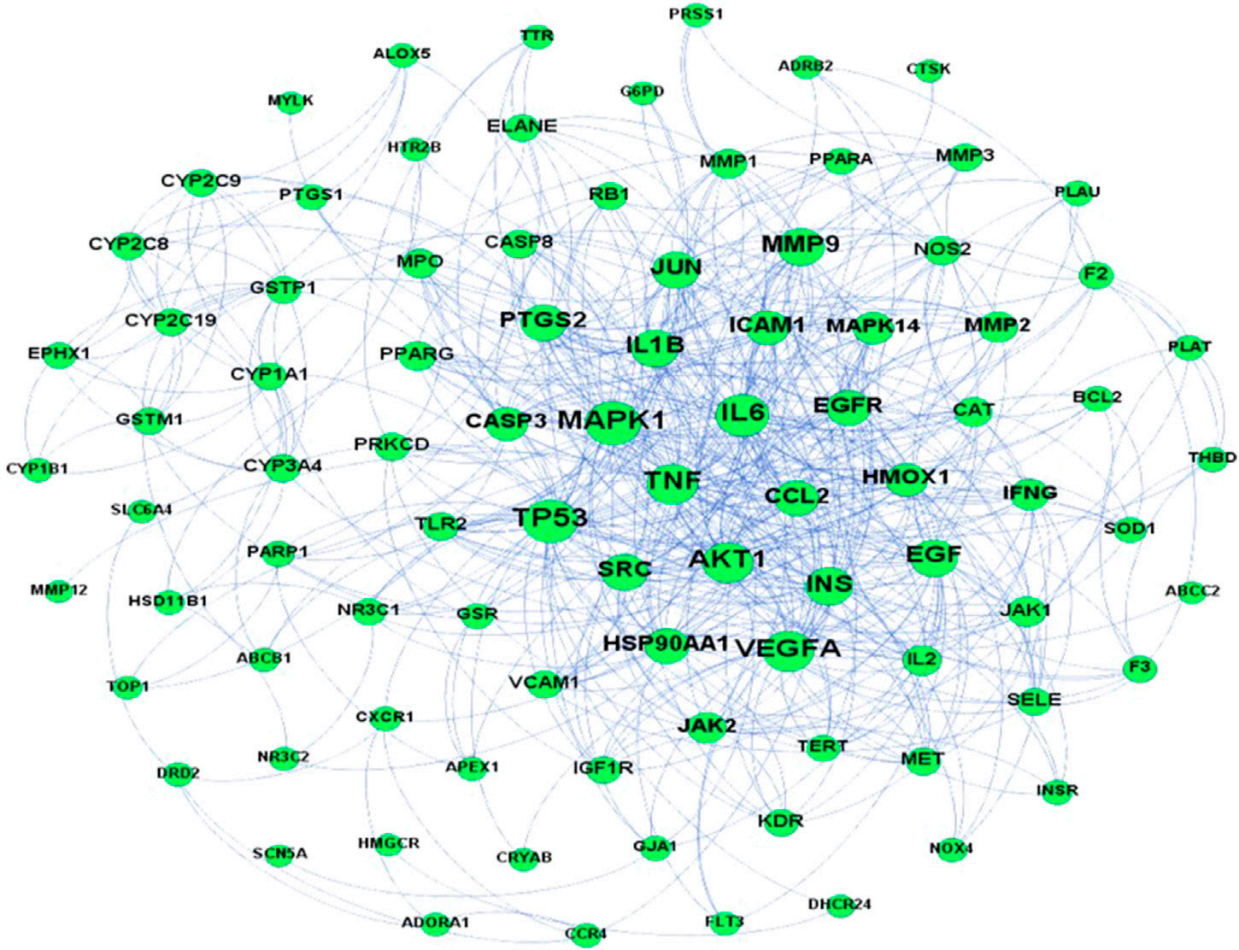
PPI network of potential pulmonary fibrosis targets acted upon by major ingredients of *H. cordata*.

A predicted PPI relationship network was obtained after the input of 101 predicted targets into the STRING platform and Cytoscape for topological analysis. MAPK1 (ERK2), TNF, AKT1, VEGFA, TP53, IL-1β, and IL-6 were advised to be the core (hub) node owing to a high network degree value, betweenness centrality, and closeness centrality in the PPI regulatory network (Figure 4).

### GO Analysis and KEGG Pathway Enrichment Analysis of Shared Targets

**FIGURE 5:**
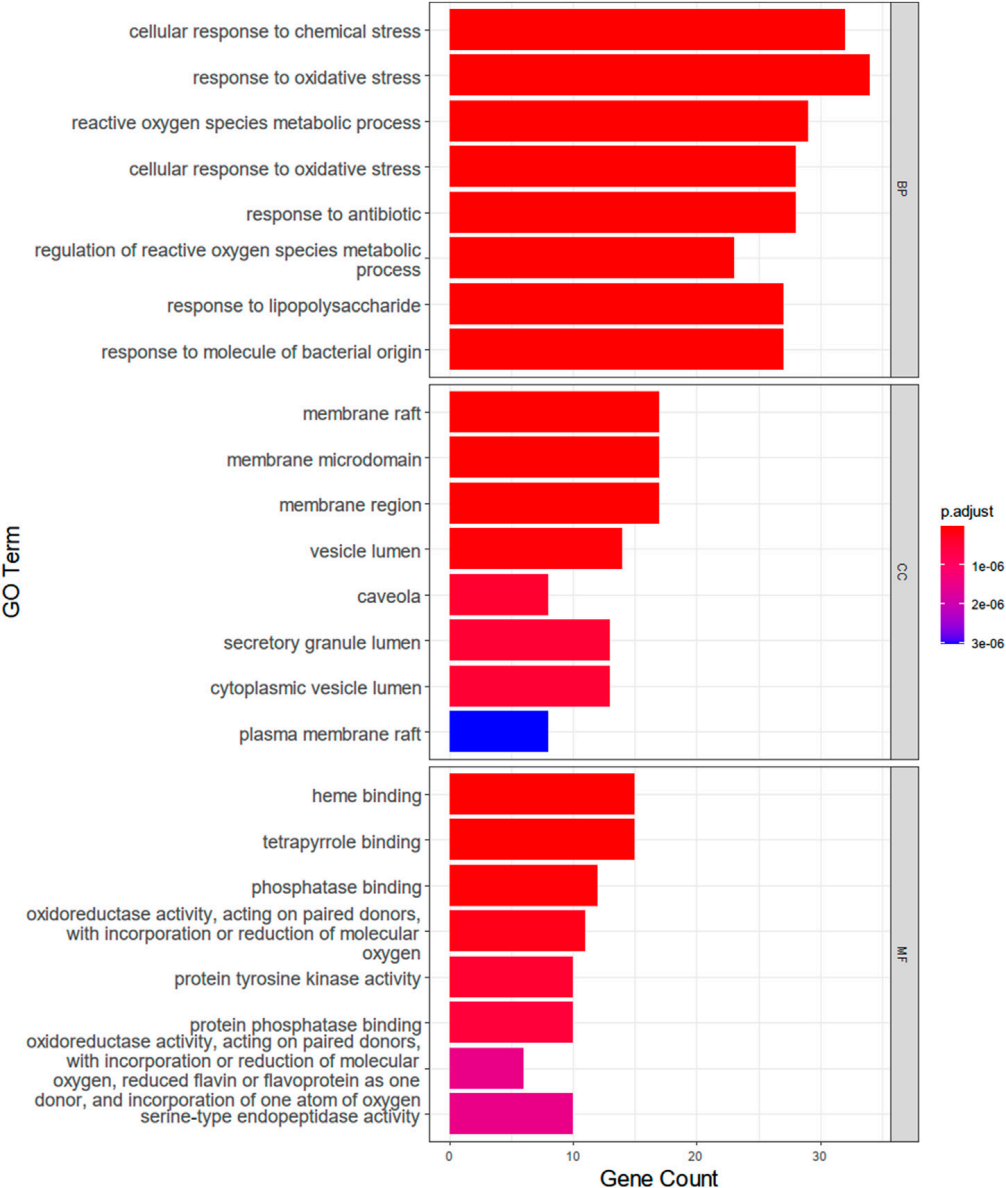
Main Go functional annotations of the action targets of major components of *H. cordata*.

**FIGURE 6:**
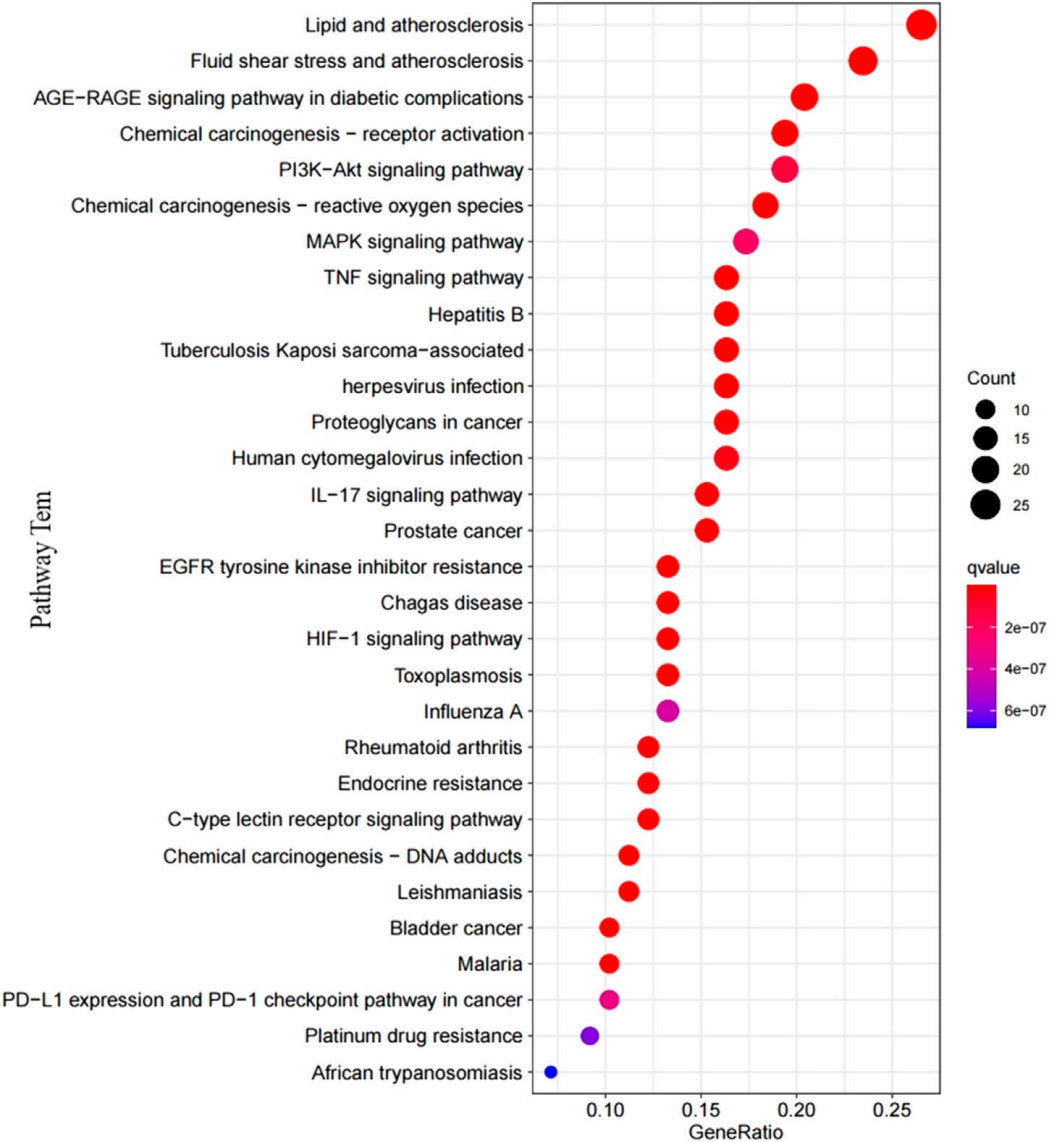
The top 30 significantly enriched terms in KEGG pathways.

**FIGURE 7:**
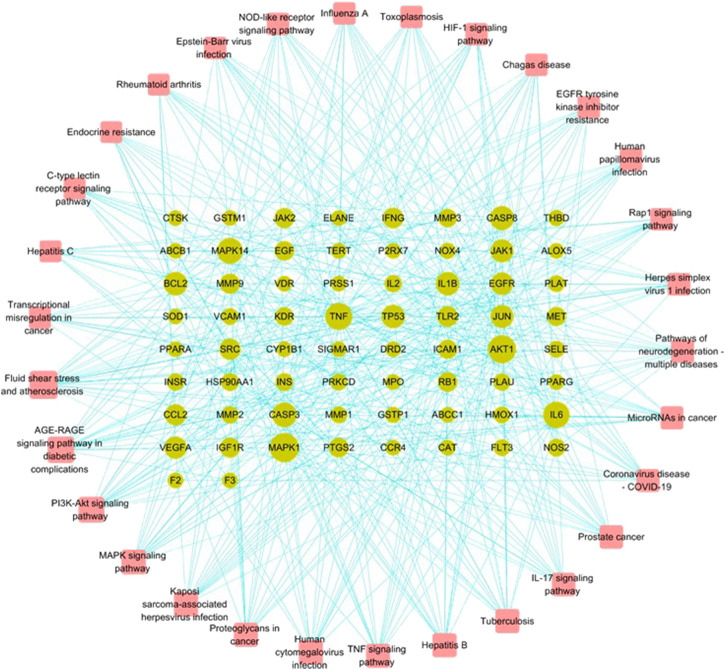
Main component action target pathway enrichment network of *H. cordata*.

With the clusterProfiler package based on R language and a filtering condition of *p* < 0.05 and q < 0.05, GO and KEGG pathway enrichment analyses of the 101 intersection targets were used to determine the biological functions of PF that were affected by *H. cordata*. GO enrichment items were composed of biological process (BP) terms, cell composition (CC) terms, and molecular function (MF) terms (Figure 5). The BP was related to the response to oxidative stress (GO: 0006979), cellular response to chemical stress (GO: 0062197), reactive oxygen species metabolic process (GO: 0072593), cellular response to oxidative stress (GO: 0034599), response to antibiotic (GO: 0046677), regulation of the reactive oxygen species metabolic process (GO: 2000377), response to lipopolysaccharide (GO: 0032496), and response to molecules of bacterial origin (GO: 0002237); in the part of CC, we obtained membrane raft (GO: 0045121), membrane microdomain (GO: 0098857), membrane region (GO: 0098589), vesicle lumen (GO: 0031983), caveola (GO: 0005901), secretory granule lumen (GO: 0034774), cytoplasmic vesicle lumen (GO: 0060205), and plasma membrane raft (GO: 0044853); in the aspect of MF, there were heme binding (GO: 0020037), tetrapyrrole binding (GO: 0046906), phosphatase binding (GO: 0019902), oxidoreductase activity, acting on paired donors, with incorporation or reduction of molecular oxygen (GO: 0016705), protein tyrosine kinase activity (GO: 0004713), protein phosphatase binding (GO: 0019903), oxidoreductase activity, acting on paired donors, with incorporation or reduction of molecular oxygen, reduced flavin or flavoprotein as one donor, and incorporation of one atom of oxygen (GO: 0016712), and serine-type endopeptidase activity (GO: 0004252). The mechanism of *H. cordata* treatment of PF may be the result of the synergistic effects of multiple pathways. 101 targets were subjected to KEGG pathway enrichment analysis, with a total of 143 pathways, and the top 30 significantly enriched KEGG pathways were identified and selected for visualization (Figure 6). The action target main enriched pathway network is shown in Figure 7.

### The Molecular Docking of Ingredient and Key Target Proteins

**FIGURE 8:**
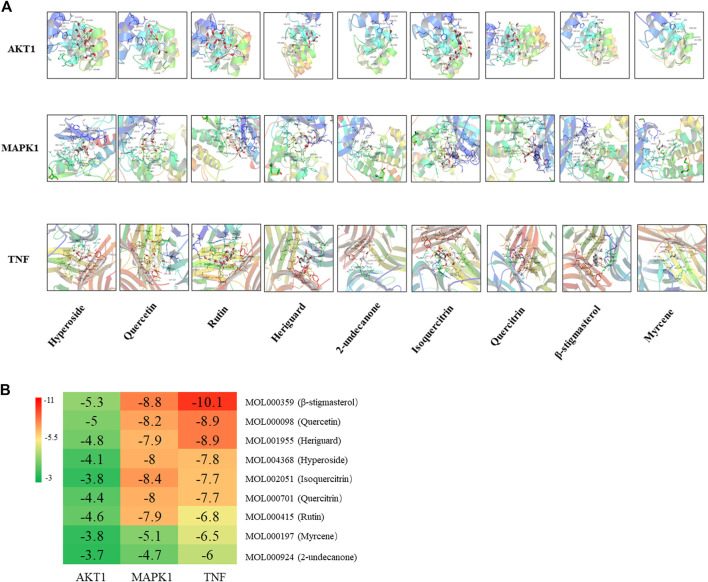
**(A)** Molecular docking results of the “bioactive compound-target gene.” **(B)** Heatmap of the docking scores of the main chemical constituents of *H. cordata* and the target proteins.

Three core target proteins (AKT1, MAPK1, and TNF-α) with high degrees were identified with nine active compounds (quercetin, hyperoside, rutin, heriguard, 2-undecanone, isoquercitrin, quercitrin, β-stigmasterol, and myrcene) by AutoDock Vina. According to the results of the molecular docking activity, the compounds selected generally had a moderate binding potential with a good medicinal reference value (Figure 8A). The docking scores were visualized using a heatmap (Figure 8B). Ultimately, three target proteins and three small molecule compounds with good docking affinity and higher content in *H. cordata* were used for subsequent experimental studies.

### Experimental Demonstration *In Vitro*

**FIGURE 9:**
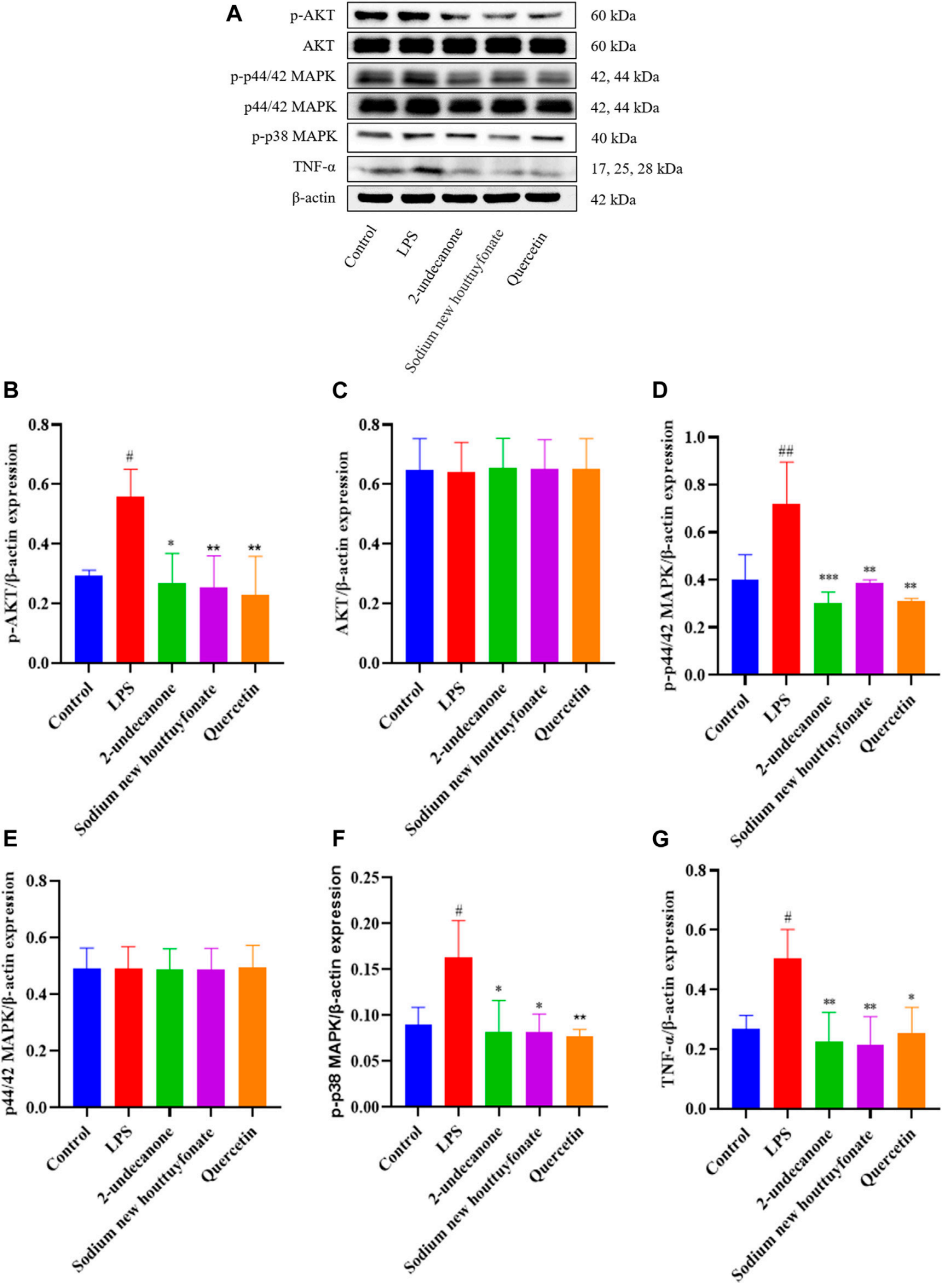
Effects of 2-undecanone, sodium new houttuyfonate, and quercetin on the protein expression of AKT, p-AKT, p44/42 MAPK (ERK1/2), p-p44/42 MAPK (ERK1/2), p-p38 MAPK, and TNF-α of LPS-induced A549 cells (A–G). #*p* < 0.05, ##*p* < 0.01, compared with the control group. *p < 0.05, **p < 0.01, compared with the LPS group.

Based on the results of network pharmacology and molecular docking, the anti-pulmonary fibrosis effect of 2-undecanone, sodium new houttuyfonate, and quercetin was necessary to be assessed with three protein-related pathways. According to the results of pre-experiment and literature data, 0.2 mM 2-undecanone, 0.3 mM sodium new houttuyfonate, and 0.05 mM quercetin were used in subsequent research. As the results were obtained, the expression of p-AKT, AKT, p44/42 MAPK (ERK1/2), p-p44/42 MAPK (ERK1/2), p-p38 MAPK, and TNF-α protein in the drug group was significantly decreased compared with that in the model group (*p* < 0.05 or *p* < 0.01) (Figures 9A–G). Our results showed that 2-undecanone, sodium new houttuyfonate, and quercetin inhibited the production of fibrosis in LPS-induced A549 cells.

## Discussion

PF is a progressive and severe condition characterized by mesenchymal cell proliferation and differentiation, ECM deposition, and remodeling of the lung parenchyma and airways. Although progress has been made in the research on PF, our understanding of its pathological mechanism remains rudimentary. There is still no complete cure for PF, and current therapies merely act to delay but not completely stop the progression of the disease.

*H. cordata*, a Chinese herb with special odor, is mainly used to treat bronchitis and upper respiratory tract infection. And studies have reported that it can inhibit rapid PF by upregulating IFN-γ and inhibiting the TGF-β1/Smad pathway (Du et al., 2012). 2-Undecanone, houttuynin, and quercetin are the main constituents of *H. cordata*. Houttuynin is the easily polymerized major constituent of the volatile oil, and the content is usually about 15%, which was first isolated from *H. cordata* in 1952 (Pan et al., 2010). Because houttuynin was unstable in chemical characteristics, sodium new houttuyfonate (sodium lauroyl-α-hydroxyethyl sulfonate, C_14_H_27_NaO_5_S, MW = 330.4), an adduct compound of houttuynin and sodium bisulfite, was developed and put in use. Sodium new houttuyfonate has better water solubility and stability and can be transformed into houttuynin under physiological conditions. Previous studies have shown that the lung is the target organ of houttuynin, and it has good therapeutic effects on pneumonia (Deng et al., 2012). Also, sodium new houttuyfonate is an effective clinical therapeutic agent applied to respiratory infection and inflammation such as acute or chronic bronchitis and pneumonia. 2-Undecanone is used as a standard marker for the quality control of *H. cordata* in the Chinese pharmacopoeia, and its content is usually about 17.0–27.5% (Yu-hong et al., 2015). Studies have shown that 2-undecanone performed various types of pharmacological functions, including anti-inflammatory and antitumor effects (Chen et al., 2014; Lou et al., 2019). Quercetin is one of the major flavonoid-active components in *H. cordata*, and the content is usually about 0.50 mg/g (Xiao-lei and Xiao-yin, 2013), which could overexpress the FasL receptor and caveolin-1 expression, reducing AKT activation and eliminating apoptosis resistance in IPF (Hohmann et al., 2019). Quercetin increases the Nrf2 activity to defend against oxidation, restore the disturbed redox balance, and reduce inflammation (Veith et al., 2017). As has been reported, quercetin can also reduce the expression of IL-6, TNF-α, and IL-1β in the bronchoalveolar lavage fluid of bleomycin-induced PF *in vivo* (Baowen et al., 2010).

Our results analyzed the ingredients in *H. cordata*, as well as the functions and potential targets against PF. According to the topological property analysis of the “drug–disease” network and the results of the PPI network analysis, the targets of the action of *H. cordata* were members of the mitogen-activated protein kinase (MAPK) family, TNF, AKT, or the cytokine VEGF. The results of the GO functional enrichment analysis revealed that *H. cordata* is mainly involved in signal transduction, inflammatory immunity, regulation of chemokine production, *etc*. The KEGG enrichment analysis showed that the possible pathways involved in the anti-pulmonary fibrosis of *H. cordata* mainly included the phosphatidylinositol-3-kinase (PI3K)/Akt signaling pathway, MAPK signaling pathway, TNF signaling pathway, IL-17 signaling pathway, HIF-1 signaling pathway, and NOD-like receptor signaling pathway. All the above signaling pathways are related to inflammation and immune responses, indicating that *H. cordata* exerts immunomodulatory and anti-inflammatory effects through multiple pathways, exactly in accordance with our previous study which shows that sodium houttuyfonate reduces BLM-induced elevation of inflammatory cytokines such as IL-1, IL-6, and TNF-α in mice (Shen et al., 2021). The PI3K/Akt signaling pathway mainly regulates cell proliferation, inflammation, and survival, and maintains the biological properties of malignant cells. PI3K, consisting of a regulatory subunit, p85, and a catalytic subunit, p110, primarily exist in the cytoplasm. Akt, also known as the protein kinase B (PKB), regulates and controls cell proliferation and apoptosis. After PI3K activation by tyrosine kinases, PIP3 was generated on the plasma membrane to interact with the PH domain of Akt, and Thr^308^ and Ser^473^ of Akt will be phosphorylated with the help of 3-phosphoinositide–dependent protein kinase 1 (PDK1) and PDK2, Akt-activated (Assinder et al., 2009). PI3K/Akt contributes to the development and progression of PF by upregulating cell growth and collagen expression (Lu et al., 2010). TNF-α, a key regulator of inflammation, could initiate and drive many pulmonary pathological diseases by inducing the accumulation of inflammatory cells, causing oxidative and nitrosative stress, airway hyperactivity, and tissue remodeling (Blaser et al., 2016). TNF-α stimulates epithelial cell proliferation and contributes to epithelial thickening and PF (Sasaki et al., 2000). MAPKs, serine–threonine protein kinases, have been proven to control cellular processes associated with fibrosis, such as myofibroblast transformation and others (Xu et al., 2004). The extracellular signal–regulated kinases 1 and 2 (ERK1/2) cascade can regulate cell proliferation, differentiation, EMT, and tumorigenesis. The activation of ERK signaling causes morphological changes and the downregulation of E-cadherin expression in epithelial cells (Grände et al., 2002). Next, molecular docking studies for MAPK1, AKT1, and TNF-α were performed using the active constituents 2-undecanone, quercitrin, β-stigmasterol, myrcene, isoquercitrin, heriguard, rutin, quercetin, and hyperoside to evaluate the thermodynamic binding effects of proteins and ligands. The results showed that the target proteins and components had good binding effects. And quercetin showed great binding strength with MAPK1, AKT1, and TNF-α, which may play an essential role in PF.

LPS, an endotoxin component of Gram-negative bacilli as an inflammatory inducer, is a vital promoter of acute lung injury (ALI) as well as promoting EMT formation in various cell types including alveolar epithelial cells (AECs) (Dong et al., 2017; Xiao et al., 2020). A549 cells were added to LPS coculture to induce EMT validating the predicted outcome of network pharmacology. Evidence showed that quercetin was well validated by its docking to IL-6, TNF, and IL-1β proteins (Yu et al., 2021). These findings strikingly supported the molecular docking and experimental results of our study that quercetin may exert anti-PF effects by targeted binding to TNF. And sodium houttuyfonate inhibited the expression of inflammatory cytokine TNF-α, which was consistent with our previous findings in bleomycin-induced pulmonary fibrosis in mice. Results from our study revealed the potential mechanism of *H. cordata* and suggested that *H. cordata* has a clinical application value in attenuating PF because of its effect in inhibiting the expression of AKT, p-AKT, p44/42 MAPK (ERK1/2), p-p44/42 MAPK (ERK1/2), p-p38 MAPK, and TNF-α *via* the PI3K/AKT, MAPK, and TNF signaling pathways. However, the mechanism needs more investigation *in vitro* and *in vivo* models.

## Conclusion

Our present study has important implications for the current understanding of the molecular mechanism of *H. cordata* in anti-pulmonary fibrosis, which involves multicomponents, multi-targets, and multi-pathways. An in-depth study should be considered by taking *H. cordata* as a novel anti-pulmonary fibrotic drug.

## Data Availability

The original contributions presented in the study are included in the article/Supplementary Material; further inquiries can be directed to the corresponding author.

## References

[B1] Assinder S. J. Dong Q. Kovacevic Z. Richardson D. R. (2009). The TGF-Beta, PI3K/Akt and PTEN Pathways: Established and Proposed Biochemical Integration in Prostate Cancer. Biochem. J. 417 (2), 411–421. 10.1042/bj20081610 19099539

[B2] Baowen Q. Yulin Z. Xin W. Wenjing X. Hao Z. Zhizhi C. (2010). A Further Investigation Concerning Correlation between Anti-fibrotic Effect of Liposomal Quercetin and Inflammatory Cytokines in Pulmonary Fibrosis. Eur. J. Pharmacol. 642 (1-3), 134–139. 10.1016/j.ejphar.2010.05.019 20510684

[B3] Blaser H. Dostert C. Mak T. W. Brenner D. (2016). TNF and ROS Crosstalk in Inflammation. Trends Cel Biol. 26 (4), 249–261. 10.1016/j.tcb.2015.12.002 26791157

[B4] Chen J. Wang W. Shi C. Fang J. (2014). A Comparative Study of Sodium Houttuyfonate and 2-undecanone for Their *In Vitro* and *In Vivo* Anti-inflammatory Activities and Stabilities. Int. J. Mol. Sci. 15 (12), 22978–22994. 10.3390/ijms151222978 25514406 PMC4284749

[B5] Cui Y. Jiang L. Yu R. Shao Y. Mei L. Tao Y. (2019). β-Carboline Alkaloids Attenuate Bleomycin Induced Pulmonary Fibrosis in Mice through Inhibiting NF-Kb/p65 Phosphorylation and Epithelial-Mesenchymal Transition. J. Ethnopharmacol 243, 112096. 10.1016/j.jep.2019.112096 31323300

[B6] Deng Z. P. Zhong D. F. Meng J. Chen X. Y. (2012). Covalent Protein Binding and Tissue Distribution of Houttuynin in Rats after Intravenous Administration of Sodium Houttuyfonate. Acta Pharmacol. Sin. 33 (4), 568–576. 10.1038/aps.2011.174 22388072 PMC4003355

[B7] Dong W. W. Zhang Y. Q. Zhu X. Y. Mao Y. F. Sun X. J. Liu Y. J. (2017). Protective Effects of Hydrogen-Rich Saline against Lipopolysaccharide-Induced Alveolar Epithelial-To-Mesenchymal Transition and Pulmonary Fibrosis. Med. Sci. Monit. 23, 2357–2364. 10.12659/msm.900452 28522797 PMC5445901

[B8] Du S. Li H. Cui Y. Yang L. Wu J. Huang H. (2012). Houttuynia Cordata Inhibits Lipopolysaccharide-Induced Rapid Pulmonary Fibrosis by Up-Regulating IFN-γ and Inhibiting the TGF-β1/Smad Pathway. Int. Immunopharmacol 13 (3), 331–340. 10.1016/j.intimp.2012.03.011 22561446 PMC7106082

[B9] George P. M. Wells A. U. Jenkins R. G. (2020). Pulmonary Fibrosis and COVID-19: the Potential Role for Antifibrotic Therapy. Lancet Respir. Med. 8 (8), 807–815. 10.1016/s2213-2600(20)30225-3 32422178 PMC7228727

[B10] Grände M. Franzen A. Karlsson J. O. Ericson L. E. Heldin N. E. Nilsson M. (2002). Transforming Growth Factor-Beta and Epidermal Growth Factor Synergistically Stimulate Epithelial to Mesenchymal Transition (EMT) through a MEK-dependent Mechanism in Primary Cultured Pig Thyrocytes. J. Cel Sci. 115 (Pt 22), 4227–4236. 10.1242/jcs.00091 12376555

[B11] Guo H. Zhang Z. Su Z. Sun C. Zhang X. Zhao X. (2016). Enhanced Anti-tumor Activity and Reduced Toxicity by Combination Andrographolide and Bleomycin in Ascitic Tumor-Bearing Mice. Eur. J. Pharmacol. 776, 52–63. 10.1016/j.ejphar.2016.02.032 26874212

[B12] He H. Tang H. Gao L. Wu Y. Feng Z. Lin H. (2015). Tanshinone IIA Attenuates Bleomycin-Induced Pulmonary Fibrosis in Rats. Mol. Med. Rep. 11 (6), 4190–4196. 10.3892/mmr.2015.3333 25672255 PMC4394983

[B13] Hohmann M. S. Habiel D. M. Coelho A. L. Verri W. A. Jr. Hogaboam C. M. (2019). Quercetin Enhances Ligand-Induced Apoptosis in Senescent Idiopathic Pulmonary Fibrosis Fibroblasts and Reduces Lung Fibrosis *In Vivo* . Am. J. Respir. Cel Mol Biol. 60 (1), 28–40. 10.1165/rcmb.2017-0289OC PMC6348716 30109946

[B14] Lau K. M. Lee K. M. Koon C. M. Cheung C. S. Lau C. P. Ho H. M. (2008). Immunomodulatory and Anti-SARS Activities of Houttuynia Cordata. J. Ethnopharmacol. 118 (1), 79–85. 10.1016/j.jep.2008.03.018 18479853 PMC7126383

[B15] Lou Y. Guo Z. Zhu Y. Kong M. Zhang R. Lu L. (2019). Houttuynia Cordata Thunb. And its Bioactive Compound 2-undecanone Significantly Suppress Benzo(a)pyrene-Induced Lung Tumorigenesis by Activating the Nrf2-HO-1/nqo-1 Signaling Pathway. J. Exp. Clin. Cancer Res. 38 (1), 242. 10.1186/s13046-019-1255-3 31174565 PMC6556055

[B16] Lu Y. Azad N. Wang L. Iyer A. K. Castranova V. Jiang B. H. (2010). Phosphatidylinositol-3-kinase/akt Regulates Bleomycin-Induced Fibroblast Proliferation and Collagen Production. Am. J. Respir. Cel Mol Biol. 42 (4), 432–441. 10.1165/rcmb.2009-0002OC PMC2848736 19520917

[B17] Meyer K. C. (2017). Pulmonary Fibrosis, Part I: Epidemiology, Pathogenesis, and Diagnosis. Expert Rev. Respir. Med. 11 (5), 343–359. 10.1080/17476348.2017.1312346 28345383

[B18] Ng L. T. Yen F. L. Liao C. W. Lin C. C. (2007). Protective Effect of Houttuynia Cordata Extract on Bleomycin-Induced Pulmonary Fibrosis in Rats. Am. J. Chin. Med. 35 (3), 465–475. 10.1142/s0192415x07004989 17597505

[B19] Pan P. Wang Y. J. Han L. Liu X. Zhao M. Yuan Y. F. (2010). Effects of Sodium Houttuyfonate on Expression of NF-Κb and MCP-1 in Membranous Glomerulonephritis. J. Ethnopharmacol 131 (1), 203–209. 10.1016/j.jep.2010.06.020 20600768

[B20] Sasaki M. Kashima M. Ito T. Watanabe A. Izumiyama N. Sano M. (2000). Differential Regulation of Metalloproteinase Production, Proliferation and Chemotaxis of Human Lung Fibroblasts by PDGF, Interleukin-1beta and TNF-Alpha. Mediators Inflamm. 9 (3-4), 155–160. 10.1080/09629350020002895 11132772 PMC1781765

[B21] Shen Y. H. Cheng M. H. Liu X. Y. Zhu D. W. Gao J. (2021). Sodium Houttuyfonate Inhibits Bleomycin Induced Pulmonary Fibrosis in Mice. Front. Pharmacol. 12, 596492. 10.3389/fphar.2021.596492 33716736 PMC7947865

[B22] Sun L. Mao M. Yan Z. Zuo C. Zhang X. (2018). A Chinese Traditional Therapy for Bleomycin-Induced Pulmonary Fibrosis in Mice. Can. Respir. J. 2018, 8491487. 10.1155/2018/8491487 30319721 PMC6167599

[B23] Veith C. Drent M. Bast A. van Schooten F. J. Boots A. W. (2017). The Disturbed Redox-Balance in Pulmonary Fibrosis Is Modulated by the Plant Flavonoid Quercetin. Toxicol. Appl. Pharmacol. 336, 40–48. 10.1016/j.taap.2017.10.001 28987380

[B24] Wang C. Song X. Li Y. Han F. Gao S. Wang X. (2013). Low-dose Paclitaxel Ameliorates Pulmonary Fibrosis by Suppressing TGF-β1/Smad3 Pathway via miR-140 Upregulation. PLoS One 8 (8), e70725. 10.1371/journal.pone.0070725 23967091 PMC3744547

[B25] Wynn T. A. Vannella K. M. (2016). Macrophages in Tissue Repair, Regeneration, and Fibrosis. Immunity 44 (3), 450–462. 10.1016/j.immuni.2016.02.015 26982353 PMC4794754

[B26] Xiao K. He W. Guan W. Hou F. Yan P. Xu J. (2020). Mesenchymal Stem Cells Reverse EMT Process through Blocking the Activation of NF-Κb and Hedgehog Pathways in LPS-Induced Acute Lung Injury. Cell Death Dis. 11 (10), 863. 10.1038/s41419-020-03034-3 33060560 PMC7567061

[B27] Xiao-lei H. Xiao-yin C. (2013). Efficient Extraction of Flavonoids from Houttuynia Cordata. Chin. Med. Mod. Distance Edu. China 11 (15), 161–162. 10.3969/j.issn.1672-2779.2013.15.108

[B28] Xu S. W. Howat S. L. Renzoni E. A. Holmes A. Pearson J. D. Dashwood M. R. (2004). Endothelin-1 Induces Expression of Matrix-Associated Genes in Lung Fibroblasts through MEK/ERK. J. Biol. Chem. 279 (22), 23098–23103. 10.1074/jbc.M311430200 15044479

[B29] Yu M. X. Song X. Ma X. Q. Hao C. X. Huang J. J. Yang W. H. (2021). Investigation into Molecular Mechanisms and High-Frequency Core TCM for Pulmonary Fibrosis Secondary to COVID-19 Based on Network Pharmacology and Data Mining. Ann. Palliat. Med. 10 (4), 3960–3975. 10.21037/apm-20-1384 33832291

[B30] Yu-hong L. Qing-miao L. Zhi-fang H. Yun-hua L. Jin-hai Y. (2015). GC Simultaneous Determination of the Contents of Seven Constituents in Volatile Oil from Houttuyniae Herba. Chin. J. Pharm. Anal. 35 (10), 1810–1814. 10.16155/j.0254-1793.2015.10.20

