# Supplementary figures and images for: Evaluating the Therapeutic Mechanisms of Selected Active Compounds in Houttuynia cordata Thunb. in Pulmonary Fibrosis via Network Pharmacology Analysis
Source: Front Pharmacol. 2021 Sep 30;12:733618. doi: 10.3389/fphar.2021.733618 (PMC8514782; doi:10.3389/fphar.2021.733618)

## Slide 1
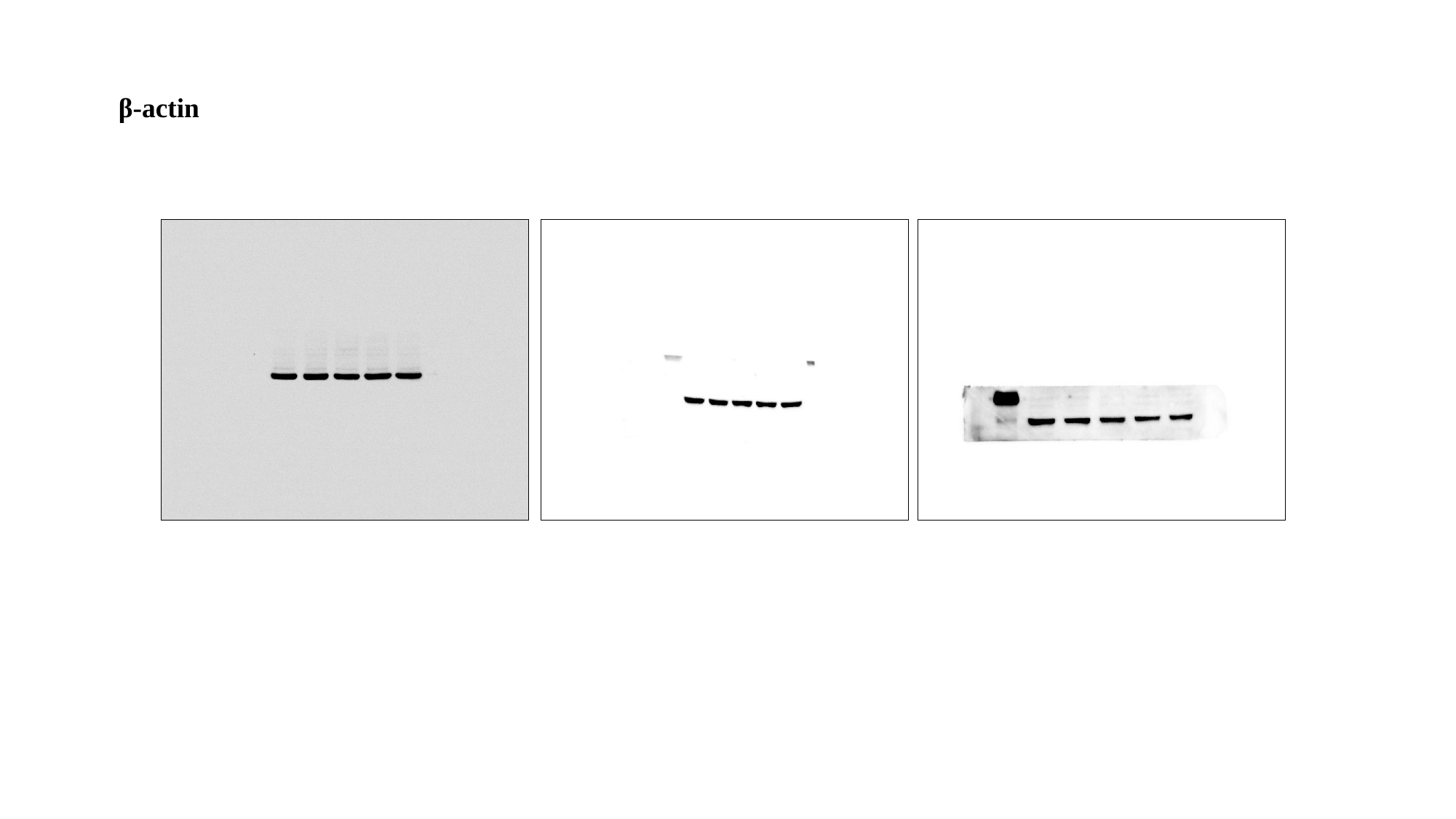

β-actin

## Slide 2
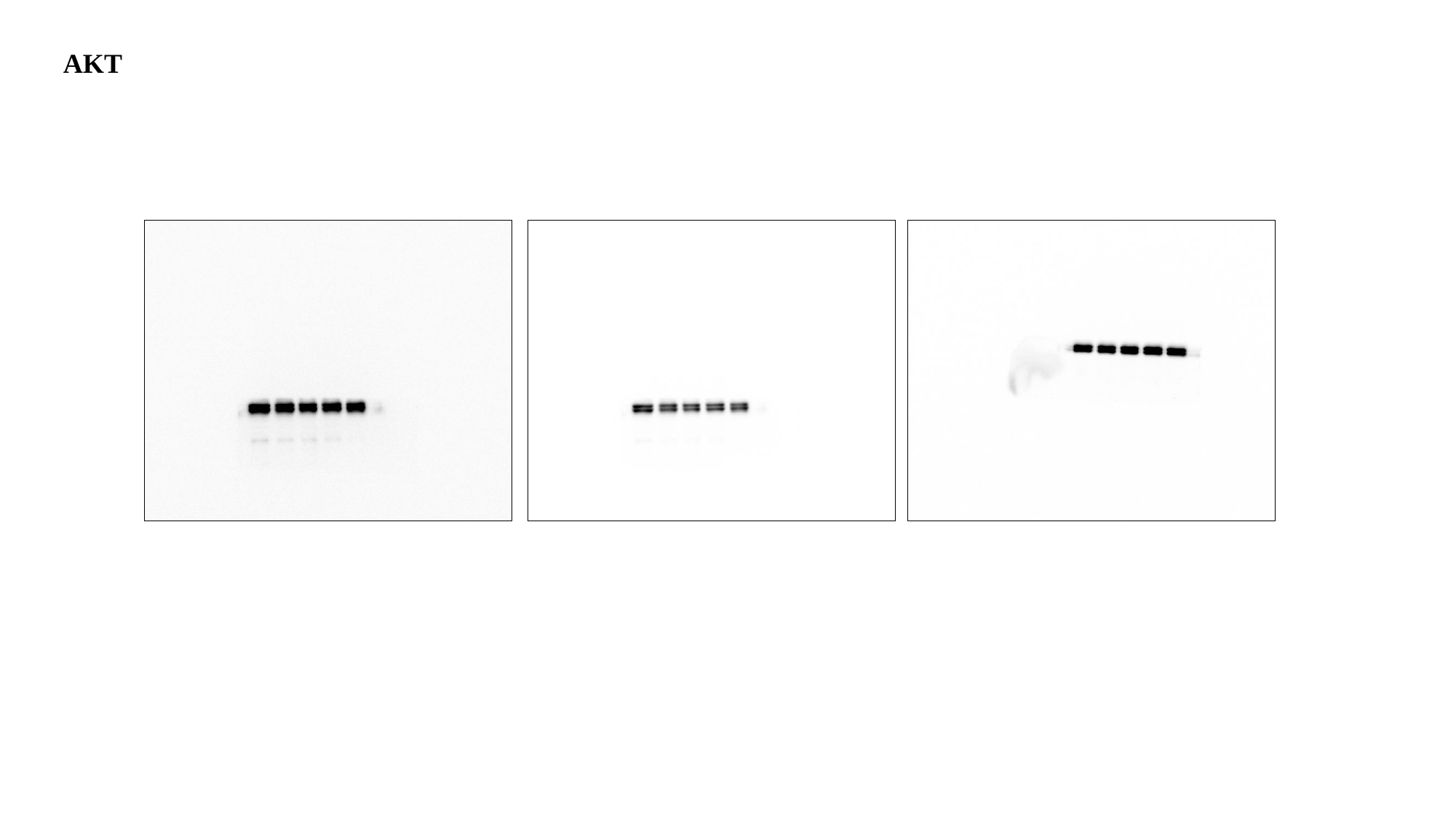

AKT

## Slide 3
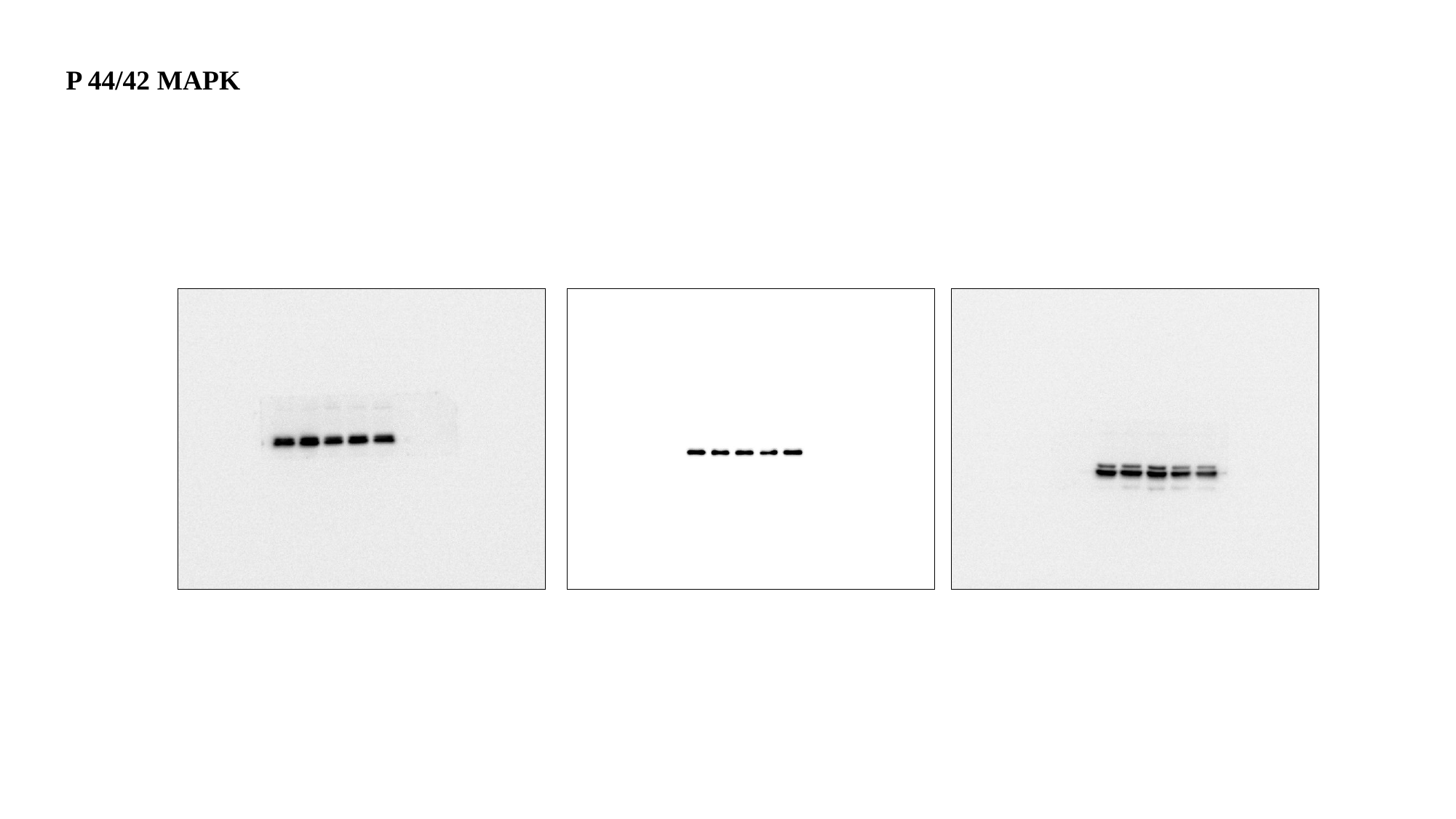

P 44/42 MAPK

## Slide 4
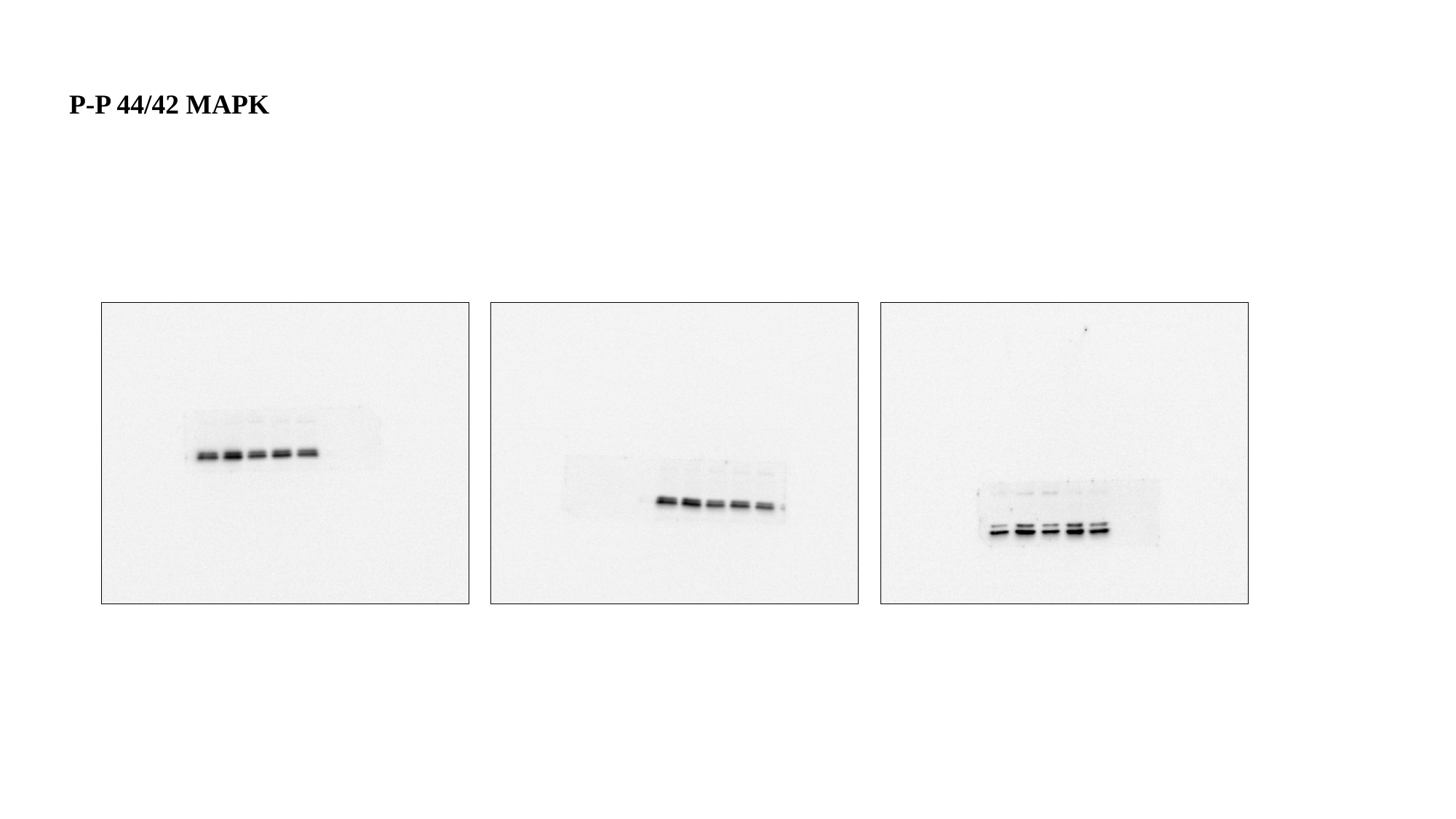

P-P 44/42 MAPK

## Slide 5
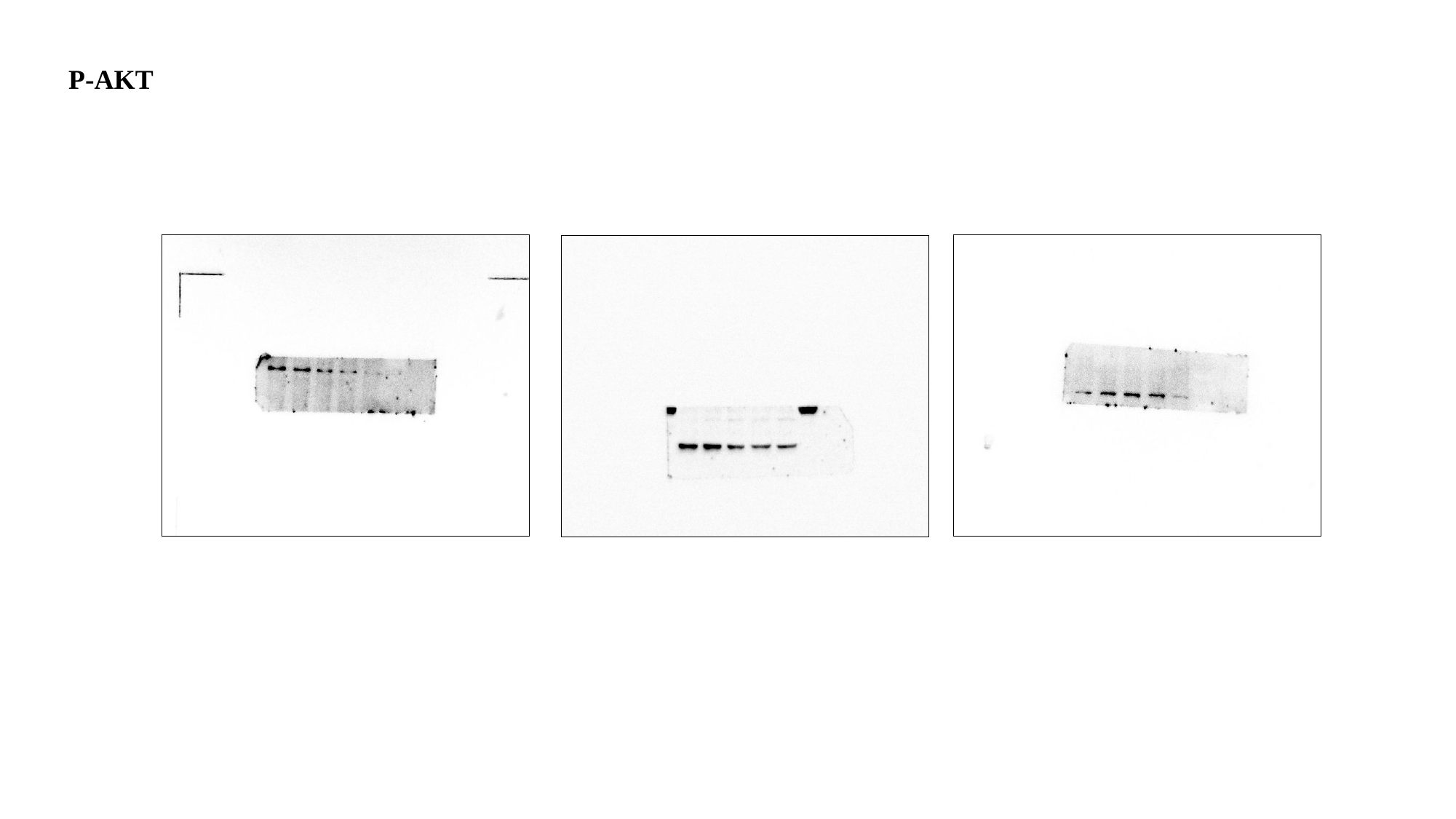

P-AKT

## Slide 6
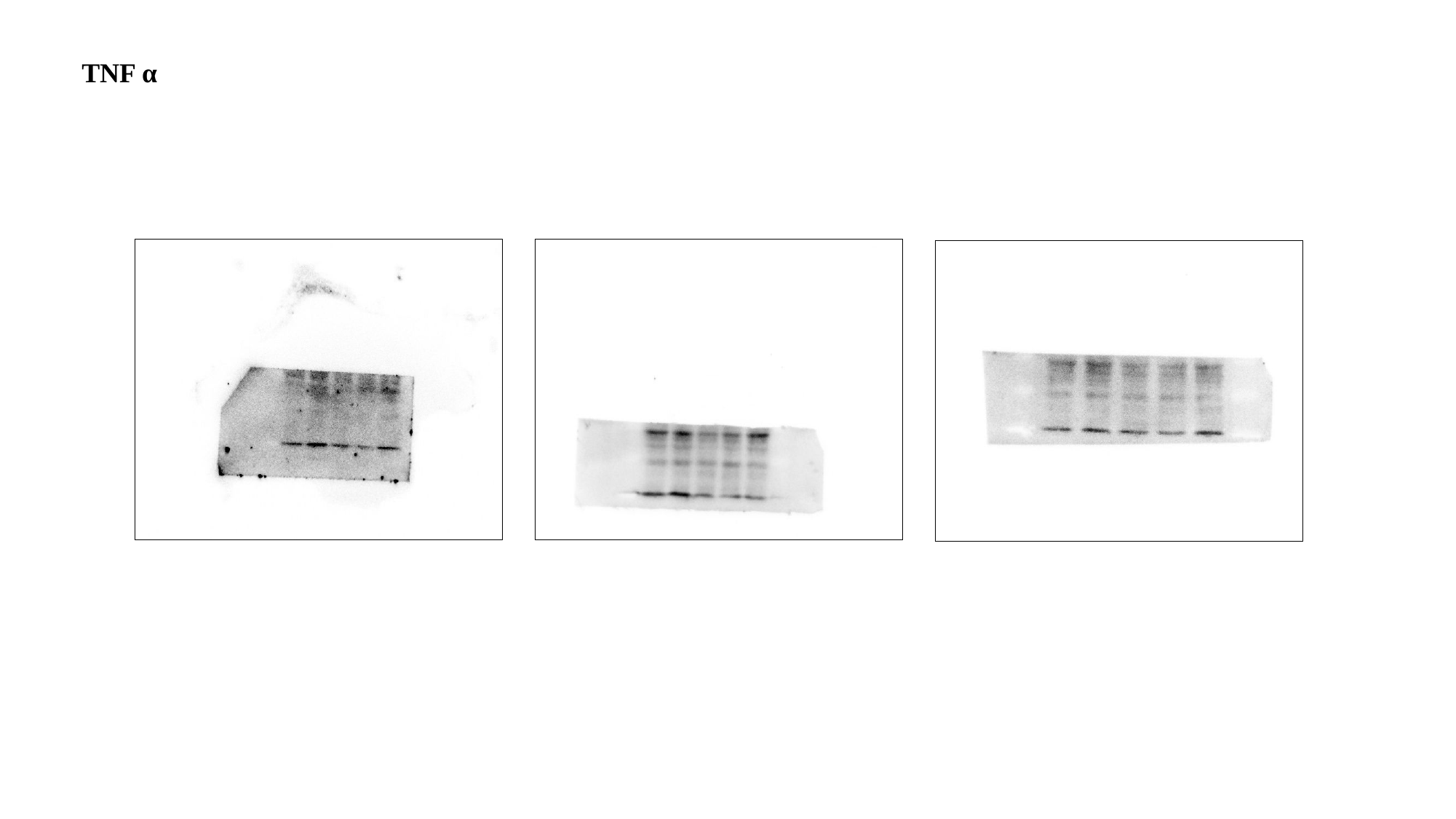

TNF α

## Slide 7
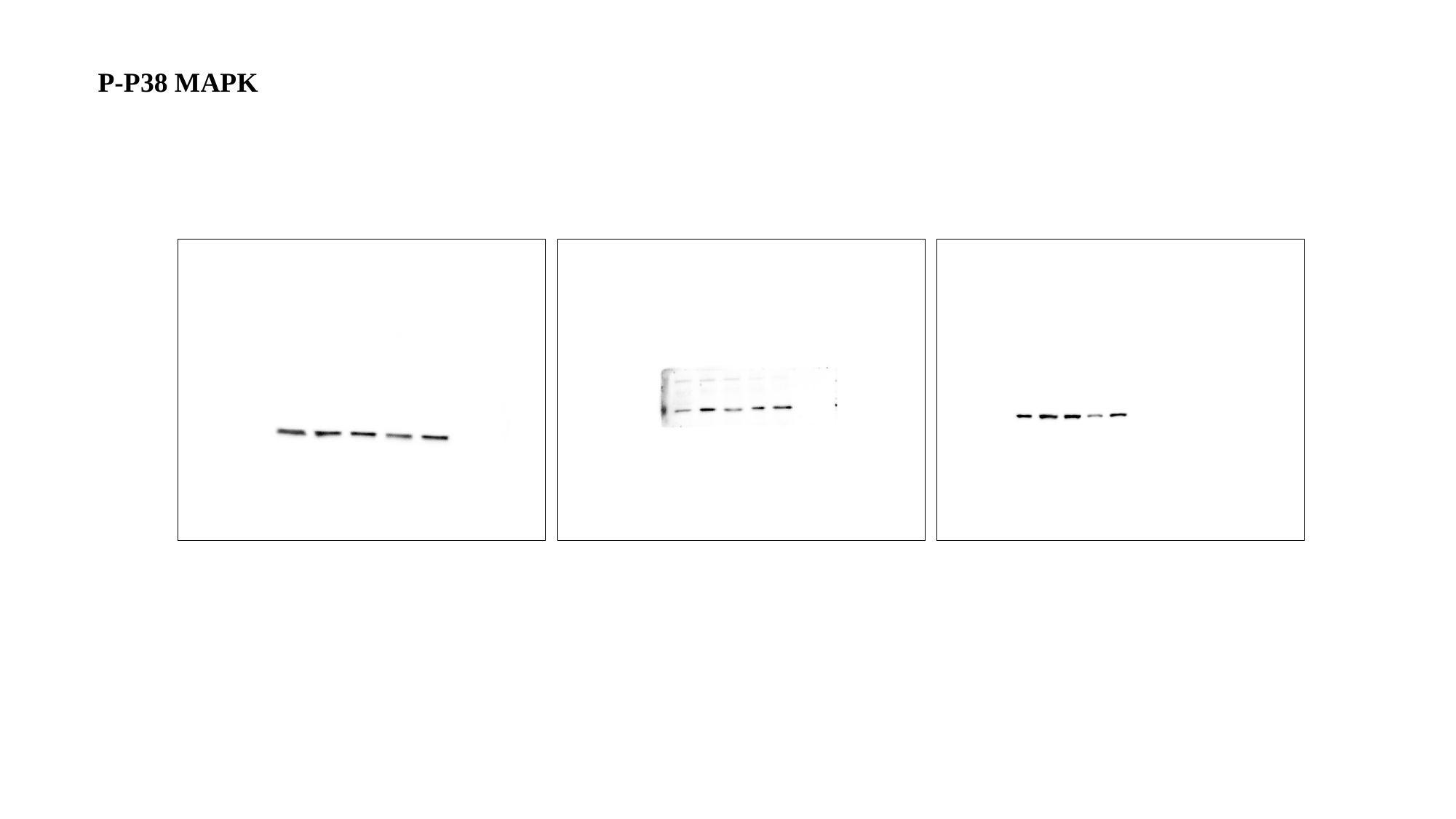

P-P38 MAPK

Supplement: Supplementary file 1 [file Presentation1.PPTX]

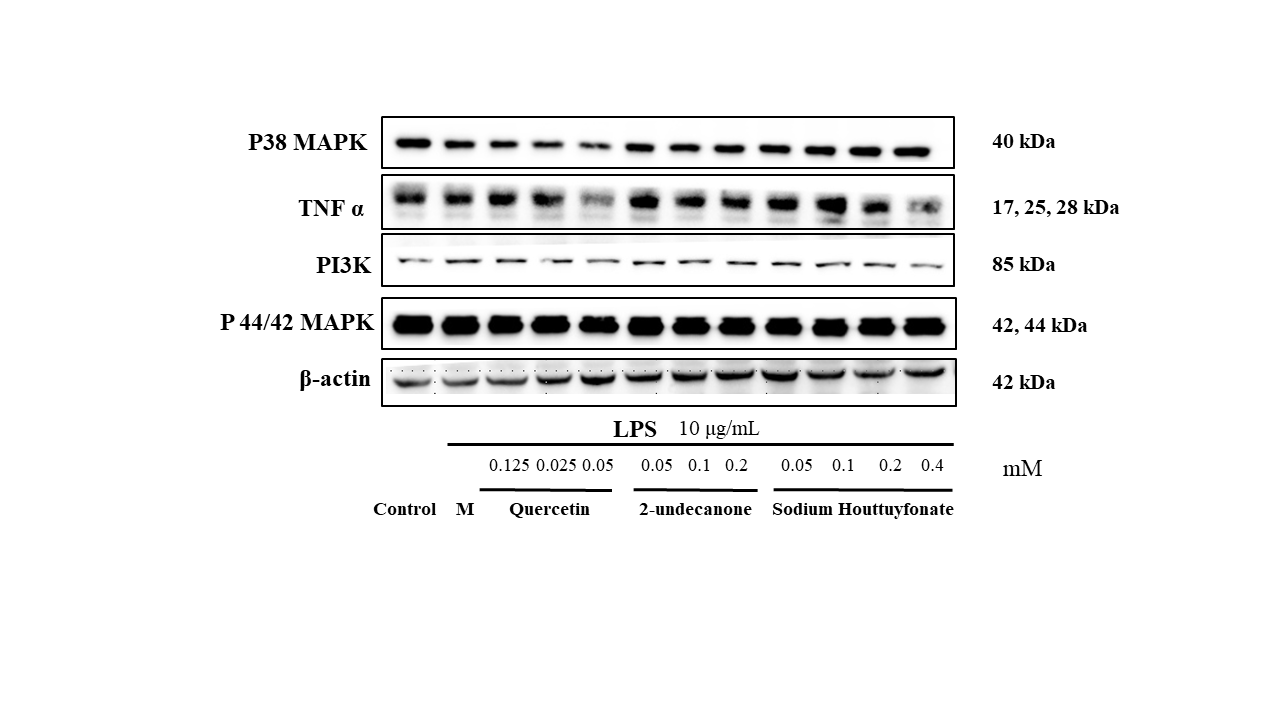

Supplement: Supplementary file 2 [file Image1.TIF]
